# Confinement Effects of a Noble Gas Dimer Inside a Fullerene Cage: Can It Be Used as an Acceptor in a DSSC?

**DOI:** 10.3389/fchem.2020.00621

**Published:** 2020-08-06

**Authors:** Debolina Paul, Harkishan Dua, Utpal Sarkar

**Affiliations:** Department of Physics, Assam University, Silchar, India

**Keywords:** noble gas dimer, fullerene, encapsulation, DFT, solar cell

## Abstract

A detailed density functional theory investigation of He_2_-encapsulated fullerene C_36_ and C_40_ has been presented here. When confinement takes place, He-He bond length shortens and a non-covalent type of interaction exists between two He atoms. Energy decomposition analysis shows that though an attractive interaction exists in free He_2_, when it is confined inside the fullerenes, repulsive interaction is observed due to the presence of dominant repulsive energy term. Fullerene C_40_, with greater size, makes the incorporation of He_2_ much easier than C_36_ as confirmed from the study of boundary crossing barrier. In addition, we have studied the possibility of using He_2_-incorporated fullerene as acceptor material in dye-sensitized solar cell (DSSC). Based on the highest energy gap, He_2_@C_40_ and bare C_40_ fullerenes are chosen for this purpose. Dye constructed with He_2_@C_40_ as an acceptor has the highest light-harvesting efficiency and correspondingly will possess the maximum short circuit current as compared to pure C_40_ acceptor.

## Introduction

Ever since the discovery of fullerene (Kroto et al., 1985), it has been studied extensively due to its fascinating properties, leading to versatile applications in various fields of nano and opto- electronics (Bhusal et al., 2016; Paul et al., 2017, 2018a,b; Qu et al., 2019), besides being used as sensors (Jaroš et al., 2019; Parey et al., 2019) and hydrogen storage devices (Chandrakumar and Ghosh, 2008; Srinivasu and Ghosh, 2012). Fullerenes are also known to exist in different isomeric forms (Małolepsza et al., 2007; Zhao et al., 2018). Insertion of various atoms and molecules into the fullerene cage has attracted a lot of interest in the scientific community and is designated as the endohedral fullerenes. It is believed that the endohedrally trapped species can manipulate different properties and affect the reactivities of the fullerene cage as well (Ravinder and Subramanian, 2011, 2012). Thus, this class of fullerenes is studied both theoretically and experimentally, having their extensive use in the field of electronics, medicine, and nanotechnology (Guha and Nakamoto, 2005; Martín, 2006; Yamada et al., 2010). They can also serve the purpose of being an acceptor in photovoltaic devices (Osuna et al., 2011). Reactivity pattern of a system (atoms and molecules) significantly changes due to confinement (Chattaraj and Sarkar, 2003; Sarkar et al., 2009, 2012; Khatua et al., 2014c; Deb et al., 2016a,b); as a result, stability increases and reduces the activation barrier. The encapsulated atoms or molecules can be either metal, non-metal, or noble gas (Ng). Noble gases, due to their closed electronic shell configuration, are generally reluctant of forming any chemical compound. However, it is possible to successfully encapsulate them inside fullerenes using techniques, such as ion bombardment (Weiske et al., 1991), molecular surgery (Saunders et al., 1994), and involving high pressure and high-temperature methods. Synthesis of the first xenon-containing molecule opened a way and interest for the researchers that noble gases are eligible to take part in chemical reactions (Bartlett, 1962). Ng-containing compound HNgY (Ng = Ar, Kr, Xe; Y = electron-withdrawing group) has been successfully synthesized by Pettersson et al. (1998). Literature survey also reveals that Feldman et al. has prepared hydrides and other Ng-related compounds (Feldman and Sukhov, 1996; Feldman et al., 1997). A number of theoretical studies on Ng have also been done apart from experimental investigations (Pan et al., 2013a,b,c, 2014a,b,c, 2015a,b,c; Saha et al., 2015; Ghara et al., 2016; Ayub, 2017). Inclusion and exclusion of Ng inside fullerene cages have been reported (Saunders et al., 1993; Hummelen et al., 1995), where an intermediate is formed by the rupture of one or more bonds, thus making an open window for the encapsulation of these noble gas atoms, although some disagreement is also set forth (Haaland et al., 2004). Experimental studies show that noble gases put inside the hollow cavity of C_60_ acquire an activation barrier energy as high as 90 kcal/mol^−1^ during its dissociation (Becker et al., 1996). Ng atoms are not only encapsulated inside larger fullerene cages (C_60_, C_70_), but they can also be successfully trapped inside much smaller cages such as C_10_H_10_, C_20_H_20_, C_32_, etc. (Darzynkiewicz and Scuseria, 1997; Jiménez-Vázquez et al., 2001; Zou et al., 2010; Chakraborty et al., 2016; Sekhar et al., 2017). Further studies on trapping two Ng atoms forming an Ng dimer (Ng_2_) inside fullerene have been first theoretically predicted by Giblin et al. (1997) and just after a year, He and Ne dimers are experimentally spotted into C_70_ fullerene (Khong et al., 1998; Laskin et al., 1998). Confinement of Ng dimers in C_60_ fullerene starting from He to Xe atoms have been studied theoretically by some groups (Krapp and Frenking, 2007; Fernández et al., 2014; Khatua et al., 2014b), where it was found that the Ng-Ng bond distances relatively get smaller when the Ng_2_ molecule is trapped inside the cage as compared to the bond distances of the isolated dimer (Krapp and Frenking, 2007). However, as a whole, these systems are thermodynamically not stable owing to the exclusion of the Ng atoms. In a study as reported by Krapp and Frenking (2007) a genuine chemical bond is found to exist between the two Xe atoms when its dimer is confined inside the C_60_ cage, while for its lighter analogs, i.e., He and Ne, the presence of weak van der Waals interaction is detected. Cerpa et al. (2009) have studied the effect of confinement on the electronic interaction between He-He bond in the host C_20_H_20_ and their results followed that a shorter He-He bond does not always indicate a chemical bond. Not only are the carbon related compounds considered as the hosts for entrapping the Ng atoms and their dimers, but hosts constituted with other elements such as B_40_ cage (Pan et al., 2018), B_12_N_12_ (Khatua et al., 2014a; Paul et al., 2019), B_16_N_16_ (Khatua et al., 2014a), ${\text{Sn}}_{12}^{2-}$, and ${\text{Pb}}_{12}^{2-}$ (Sekhar et al., 2017) are also reported. Encapsulation of Ng and Ng_2_ in B_12_N_12_ and B_16_N_16_ fullerenes show that the dimer He_2_ undergoes not only vibration and translation, but also rotation inside the cage. These investigations on noble gases encourage further comprehensive study on their confinement with different host materials.

Fullerenes possessed with electron-deficient characteristics are used as electron acceptors in a solar cell device (Liu and Troisi, 2011; Eom et al., 2014; Leng et al., 2014; Shimata et al., 2016). Dye-sensitized solar cell (DSSC) (O'Regan and Grätzel, 1991) is one of the widely used organic solar cells because it offers the possibility to convert light photons to electrical energy at a low cost. A dye in a DSSC consists of three parts: an electron-rich donor, a spacer, and an electron-deficient acceptor. Among the acceptors, [6,6]-phenyl-C_61_-butyric acid methyl ester (PCBM) has been widely investigated (Liu and Troisi, 2011). Apart from fullerene C_60_ in PCBM, C_70_ cage is also used (Leng et al., 2014). Endohedral fullerenes can be used in designing solar cells (Ross et al., 2009) where the confined atoms are supposedly contributing to the efficiencies of the carrier transport (Yamada et al., 2010).

Inspired by all these works on noble gas, herein, we have investigated the influence of He_2_ molecule when trapped inside C_36_ and C_40_ fullerenes by calculating the energetic stability, bonding analysis, energy decomposition analysis, distortion energy, and boundary-crossing barrier energy of He_2_@C_X_ (*X* = 36, 40). A glimpse of the UV-visible absorption spectra of He_2_@C_X_ is presented. Finally, considering the energetically most stable fullerene among the two (He_2_@C_40_), various parameters associated with the study of a dye-sensitized solar cell have been investigated. To make a comparison, pristine C_40_ fullerene is considered for this particular case study.

## Computational Details

All the structures have been optimized using density functional theory (DFT) methodology as implemented in Gaussian 09 program package (Frisch et al., 2009), using M05-2X functional (Hohenstein et al., 2008). We have used 6-31+g(d,p) basis set for the C atoms and def2-TZVP basis set for the He atoms.

Dissociation energy (*D*_*e*_) and distortion energy (*E*_*d*_) are calculated using the relations:

(1) $$\begin{array}{r} D_{e}=\left[\left(E_{C_{X}}+E_{He_{2}}\right)-E_{He_{2}@C_{X}}\right] \end{array}$$

(2) $$\begin{array}{r} E_{d}=E_{C_{X}\left(expanded\right)}-E_{C_{X}} \end{array}$$

where *E*_*C*_*X*__ is the total energy of pristine *C*_*X*_ fullerenes, *E*_*H*_*e*__2_@*C*_*X*__ is the total energy of the He_2_ encapsulated fullerenes (i.e., He_2_@C_36_ and He_2_@C_40_), *E*_*C*_*X*_(*expanded*)_ is the energy calculated by removing the He_2_ dimer and evaluating single point energy of the expanded *C*_*X*_ fullerenes, and *E*_*H*_*e*__2__ is the energy of He_2_ dimer.

The boundary-crossing barrier of the He_2_ dimer through one hexagonal face of the fullerenes has been obtained by scanning the potential energy surface at different distances from the center of the fullerene cages. GAMESS software (Schmidt et al., 1993) has been implemented in calculation of energy decomposition analysis. Multiwfn software (Lu and Chen, 2011) is used to perform the topological analysis of electron density. Time-dependent density functional theory (TDDFT) has been employed to check the absorption spectra for the first 40 excited state transitions of the fullerenes and GaussSum software (O'Boyle et al., 2008) has been used for plotting. For calculating solar cell parameters, we have considered B3LYP functional and 6–31 g basis set for all the atoms.

## Results and Discussion

**Figure 1:**
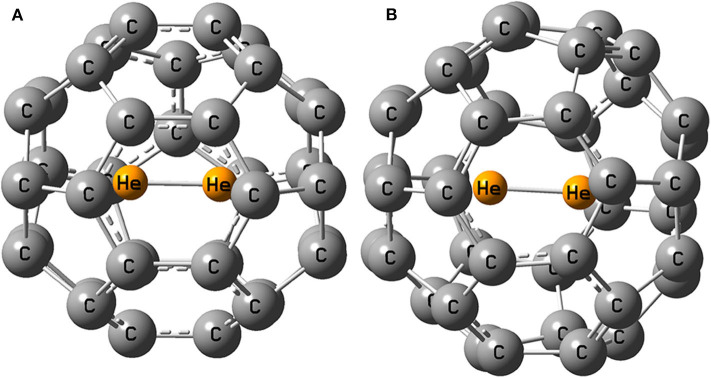
Optimized geometry of **(A)** He_2_@C_36_ and **(B)** He_2_@C_40_.

The optimized structure of He_2_ dimer encapsulated fullerenes are shown in Figure 1 below.

Upon optimization, He_2_ dimer orients itself in the middle of the fullerene cage in such a way that it can move through the midpoint of a six-membered ring easily when put inside C_36_ and C_40_ cages. In the beginning, empty C_36_ and C_40_ fullerenes (Figure S1) possess *D*_2d_ and *D*_2_ symmetry, respectively. On enclosing He_2_ inside them, the symmetry of C_36_ (i.e., He_2_@C_36_) changes to *C*_2_ but C_40_ (i.e., He_2_@C_40_) successfully retains its original symmetry *D*_2_. The highest dissociation energy is found when He_2_ is encapsulated inside C_36_ cage (−53.1 kcal/mol) and naturally, the least is found for C_40_ fullerene, having a value of −45.6 kcal/mol. Negative values of dissociation energy reveal that He_2_ encapsulation in them is not favorable. It should be noted that cage distortion and Pauli repulsion plays an important role in destabilization. However, with the increase in the size of the fullerene cage (C_36_ < C_40_), destabilization decreases due to enlargement of space inside the cavity. Hence, it may be inferred that larger cages may lead toward favoring He_2_ encapsulation in them. This result is also supported by the values of energy gap of He_2_-incorporated C_36_ and C_40_ fullerenes. The larger fullerene C_40_ records a value of 3.980 eV, whereas C_36_ has 3.028 eV energy gap when they both hold the He_2_ dimer inside them, indicating that He_2_@C_40_ is more stable than He_2_@C_36_ owing to its greater energy gap in comparison.

### Distortion Energy

The encapsulation of the He_2_ dimer inside the fullerene cages expands the surface area of the fullerene cage and this expansion may increase the binding energy of the system. The calculated distortion energy for He_2_@C_36_ is 1.6 kcal/mol and for He_2_@C_40_ is 1.7 kcal/mol. Thus, He_2_ dimer has to expend this amount of energy barriers to get encapsulated inside the fullerene cages, as can be seen from the distortion energy values.

### Structural Properties and Bonding Analysis

In order to get an idea about how the confinement of He_2_ affects itself as well as the fullerene encaging it, we have calculated the bond lengths of the He_2_ dimer both in unconfined and confined state. We have also performed electron density analysis, which will further strengthen the results. The equilibrium bond length for an isolated He_2_ is calculated to be 2.666 Å, which is exactly same as that of Khatua et al. (2014a). As expected, confinement reduces the bond length of He_2_ dimer, which is found from the investigation. More specifically, when He_2_ is entrapped inside C_36_, its bond length diminishes to 1.520 Å, while with the larger C_40_ cage, it has a value of 1.546 Å.

**Figure 2:**
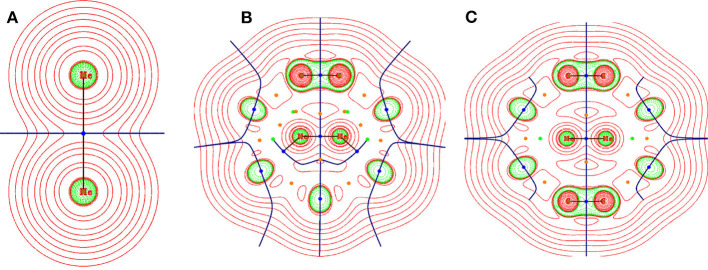
Contour plot representation of Laplacian of electron density at a particular plane of **(A)** He_2_, **(B)** He_2_@C_36_, and **(C)** He_2_@C_40_. Red region depicts the area having ∇^2^ ρ(r_c_) > 0 and green region depicts the area having ∇^2^ ρ(r_c_) < 0.

To understand the nature of interaction between the two He atoms both inside and outside fullerene C_36_ and C_40_ and surrounding C atoms when He_2_ is placed inside the cages, the electron density analysis at the bond critical point (BCP) was performed (Deb et al., 2018) by employing the Bader's atoms-in-molecules theory (Bader, 1990). The corresponding topological descriptors are tabulated in Table 1 and the contour plot of the Laplacian of electron densities at the BCPs are shown in Figure 2. At the BCP, depletion and accumulation of electron density [ρ(*r*_*c*_)] can be well understood from the positive and negative values of the Laplacian of electron density $\left(\nabla^{2}\rho_{BCP}\right)$, respectively. Thus, when the magnitude of $\nabla^{2}\rho_{BCP}<0$, a covalent or shared bond is formed between two atoms while a non-covalent bond is expected when ${\;\nabla }^{2}\rho_{BCP}>0$. This criterion is helpful in explaining the bonding nature in many systems, but for the systems involving heavier atoms or with 3d orbitals, considering only $\left[\nabla^{2}\rho \left(r_{c}\right)\right]\;$ is not adequate to describe the nature of bonding connecting the heavier atoms. Therefore, some more topological descriptors like the local kinetic energy density [*G* (*r*_*c*_)], local potential energy density [*V* (*r*_*c*_)], and local electron energy density [*H*(*r*_*c*_)], including two ratios −*G* (*r*_*c*_)/*V* (*r*_*c*_) and *G* (*r*_*c*_)/ρ(*r*_*c*_), become essential to knowing the bonding nature. Based on these parameters, Cremer and Kraka (1984) proposed that if ∇^2^ρ(r_c_) > 0 and H(r_c_) < 0, then the nature of bonding is partially covalent. It is also stated that if the ratio −*G* (*r*_*c*_)/*V* (*r*_*c*_) falls in the range 0.5–1, then there exists some degree of covalent character (partial covalent type) and if −*G* (*r*_*c*_)/*V* (*r*_*c*_) > 1, a purely non-covalent type of interaction is formed. Further, the magnitude of *G* (*r*_*c*_)/ρ(*r*_*c*_) < 1 is also indicative of the presence of covalency in any bond.

**Table 1:** Electron density descriptors (in au) at the bond critical points (BCPs) in between some selected bonds of He_2_, He_2_@C_X_ (*X* = 36, 40).

**System**	**BCP**	**ρ(*r*_*c*_)**	**$\nabla^{2}\rho (r_{c})$**	***G*(*r*_*c*_)**	***V*(*r*_*c*_)**	***H*(*r*_*c*_)**	**−*G*(*r*_*c*_)/*V*(*r*_*c*_)**	***G*(*r*_*c*_)/ρ(*r*_*c*_)**
He_2_	He(1)-He(2)	0.002	0.012	0.002	−0.001	0.001	−2.000	1.000
He_2_@C_36_	He(37)-He(38)	0.045	0.276	0.066	−0.062	0.003	−1.065	1.467
	He(37)-C(6)	0.026	0.142	0.031	−0.026	0.005	−1.192	1.192
He_2_@C_40_	He(41)-He(42)	0.042	0.257	0.060	−0.055	0.005	−1.091	1.429
	He(41)-C(20)	0.025	0.133	0.029	−0.024	0.005	−1.208	1.160

Present case deals with two types of bonds, i.e., one between He-He (in He_2_ and He_2_@C_36_, He_2_@C_40_) and another between He-C (in He_2_@C_36_ and He_2_@C_40_); corresponding labeled figure is provided in Figure S2. From Table 1, it is seen that both ∇^2^ρ(r_c_) and H(r_c_) have values > 0, which means that a non-covalent type of bonding exists between them. The ratios −*G* (*r*_*c*_)/*V* (*r*_*c*_) and *G* (*r*_*c*_)/ρ(*r*_*c*_) both have a value > 1, which again emphasizes the finding that a non-covalent type of interaction is present between both He-He and He-C bonds. It should be mentioned here that along with positive ∇^2^ρ(r_c_), the magnitude of ρ(*r*_*c*_) at the BCP is also lower than 0.1 au, which is the threshold value for ρ(*r*_*c*_) considered here, and again confirms that the studied bonds are not of covalent type. Now examining the He-He bonds in the two host fullerenes C_36_ and C_40_, one can find that the ρ(*r*_*c*_) value of He-He bond present in C_36_ is the highest between the two hosts, recording a value of 0.045 au. However, it may be noted that for unconfined He_2_, the values of ρ(r_c_), ∇^2^ρ(r_c_), *G* (*r*_*c*_), *V* (*r*_*c*_), and *H* (*r*_*c*_) are very small and almost close to zero, but when it gets confined inside the cages, the values are considerably increased. This indicates that confinement of He_2_ inside fullerene C_36_ and C_40_ has affected its various parameters.

### Energy Decomposition Analysis

To examine the interdependence of He_2_ inside C_36_ and C_40_ fullerene cages, we studied the interaction energy and the different contributing energy terms in connection with the total interaction obtained from all-electron calculations using localized molecular orbital energy decomposition analysis (LMO-EDA). To make a comparison, we also checked the interaction energy for an unconfined He_2_ dimer. The interaction energy (*E*_*int*_) can be decomposed into five energy components: electrostatic (*E*_*elec*_), exchange (*E*_*exc*_), repulsion (*E*_*rep*_), polarization (*E*_*pol*_), and dispersion (*E*_*disp*_). This particular study will shed light into the attractive (negative *E*_*int*_) or repulsive (positive *E*_*int*_) nature of interaction between the He_2_ molecule and the concerned fullerene cages. For the He_2_-entrapped C_36_ and C_40_ fullerenes, He_2_ molecule is considered as one fragment and the associated fullerene as the other. Here, the electrostatic energy represents the classical Coulomb interaction, the exchange energy includes the Pauli repulsion. The repulsion energy deals with the contribution coming from the exchange of parallel spin electrons between the two fragments. The energy gained due to the orbital relaxation of one fragment because of the existence of other fragment having undistorted charge distribution in the former fragment's proximity is accounted for the polarization energy. Lastly, the dispersion energy comes from the instantaneous correlation of fluctuating electron density distribution between the two fragments. From Table 2, we may find that attractive nature of interaction is found when two He atoms form a He_2_ dimer, which is clear from its negative interaction energy. However, the situation is changed as soon as the dimer gets confined inside the two fullerene cages and records a positive value of interaction energy. In case of free He_2_ dimer, all the energy components are less in magnitude, with zero contribution coming from the polarization energy. Now, coming to the confined He_2_ inside the cages, it can be seen that *E*_*elec*_, *E*_*exc*_, *E*_*pol*_, and *E*_*disp*_ energies are attractive in nature. Of all the attractive terms, *E*_*exc*_ contributes the most toward the total attraction energy, the second leading contributor being *E*_*disp*_ for both the systems. The electrostatic energy makes the third-highest attractive contribution and the polarization term with a smaller value (as compared to other attractive terms) is put in the last of all. However, the Pauli repulsion energy component, *E*_*rep*_, is so repulsive with the highest magnitude (positive) among all other energy terms that it makes the net interaction energy term positive and thus overall repulsive interaction energy is found for He_2_ incorporated fullerenes. The highest repulsive energy is found for He_2_@C_36_ (149.05 kcal/mol), whereas He_2_@C_40_ records much less repulsive energy than He_2_@C_36_, with a value 120.18 kcal/mol. It may be noted here that the steep rise in the destabilizing Pauli repulsive energy term in the case of He_2_-entrapped C_36_ and C_40_ fullerene and a positive interaction energy is due to the compression of bond distance in He_2_ dimer confined inside the cages in comparison to the isolated He_2_ dimer. One important finding is that when compared to He_2_ entrapped in B_12_N_12_ and B_16_N_16_ cages (Khatua et al., 2014a), the total interaction energy is much lowered in the present study owing to the increase in the size of the fullerene cages. This may indicate that larger cages can accommodate He_2_ dimer easily and may at some point stabilize the whole system.

**Table 2:** Energy decomposition analysis of He_2_ and He_2_@C_X_ (*X* = 36, 40).

**System**	***E*_*int*_**	***E*_*elec*_**	***E*_*exc*_**	***E*_rep_**	***E*_*pol*_**	***E*_*disp*_**
He_2_	−0.02	−0.02	−0.07	0.20	−0.00	−0.12
C_36_@He_2_	28.98	−25.02	−48.98	149.05	−7.54	−38.52
C_40_@He_2_	21.29	−19.74	−38.21	120.18	−5.89	−35.06

*All the values are in kcal/mol*.

### Barrier Crossing Energy

**Figure 3:**
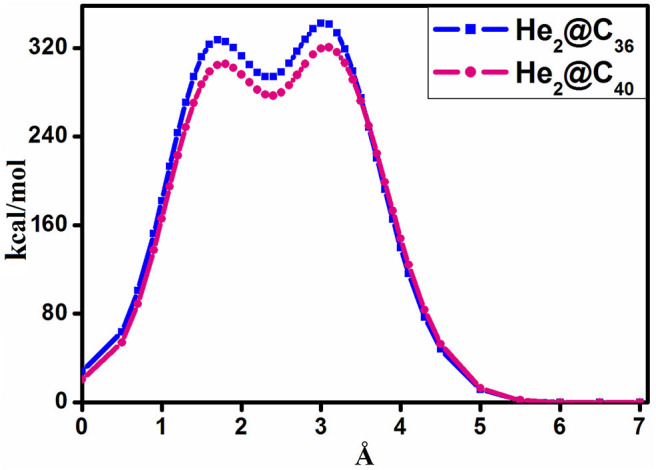
Boundary-crossing barrier of He_2_@C_X_ (*X* = 36, 40).

**Figure 4:**
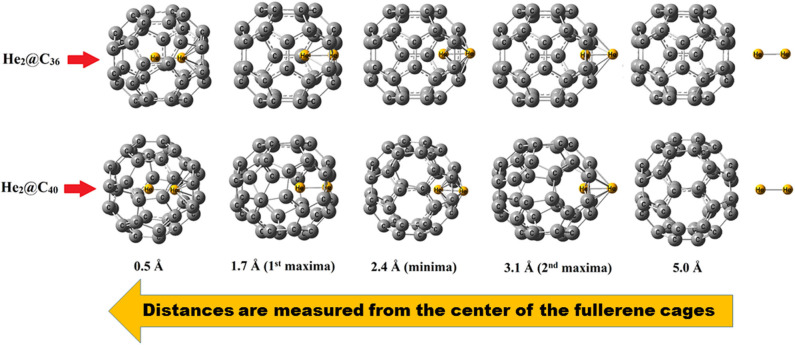
Different pictures during the encapsulation of He_2_ inside fullerene C_36_ and C_40_.

The barrier crossing energy of He_2_@C_36_ and He_2_@C_40_ fullerenes have been depicted in Figure 3. The potential of an atom or a molecule to enter/exit into/from a fullerene cage is measured by speculating the boundary-crossing barriers. The corresponding energies provide information about the permeability and kinetic barrier of the atom or the molecule for crossing the boundary of the fullerene cage. Thus, to observe the movement of He_2_ through C_36_ and C_40_ fullerenes, the boundary-crossing barrier has been studied. Also, five pictures of He_2_ encapsulation in fullerenes are shown in Figure 4. Now, since an easy translation of the dimer into the cage can be done through a six-membered ring (instead of five-membered ring), the scanning process for its barrier crossing is calculated through a hexagon only. From Figure 3, we may observe that there are two peaks for each of the curves, with the second one (2nd maxima) from left much steeper than the first one (1st maxima). The barrier crossing energy is calculated in such a way that at the beginning the He_2_ dimer is put at a distance of 7 Å from the center and then it is moved toward the cage to finally get placed in the center of the fullerene cage. The energy along this path is noted and the resulting curve is shown in Figure 3. The presence of two peaks is due to the two He atoms, which enter through the cage. The first peak from right (2nd maxima) corresponds to the energy when the first He atom passes through the cage, then the energy comes down slightly corresponding to the valley between two peaks, where the two He atoms are equally placed, one inside and another outside the surface of the cage. After that, when the second He atom just reaches the surface of the fullerene cage, the corresponding energy again rises (1st maxima), but with a relatively lower magnitude than that of the former peak value. Thereafter the energy value gradually decreases as the He_2_ dimer reaches the center of the cage, where it takes the minimum energy position. Among the two cages, namely fullerene C_36_ and C_40_, it is clear from Figure 3 that He_2_ dimer possesses lower barrier crossing energy when it enters the C_40_ fullerene as compared to C_36_. Thus, it is obvious that with increase in the size of the cage, it becomes comparatively easier for the He_2_ dimer to be encapsulated inside it. Moreover, high energy barrier for the encapsulation process of any atom/molecule to be confined inside any closed complex suggests their kinetic stability, and once these encapsulated clusters are formed, they cannot dissociate into fragments owing to higher kinetic barrier (Sekhar et al., 2017). Here in this study, both the He_2_ encapsulated fullerenes, He_2_@C_36_ (327. 3 kcal/mol at 1st maxima and 342.2 kcal/mol at 2nd maxima) and He_2_@C_40_ (305.4 kcal/mol at 1st maxima and 320.4 kcal/mol at 2nd maxima), have a much higher energy barrier when the two He atoms of the constituting He_2_ dimer cross the surface of the fullerene cages. Thus, they may be considered as kinetically stable systems.

### Absorption Spectra

**Figure 5:**
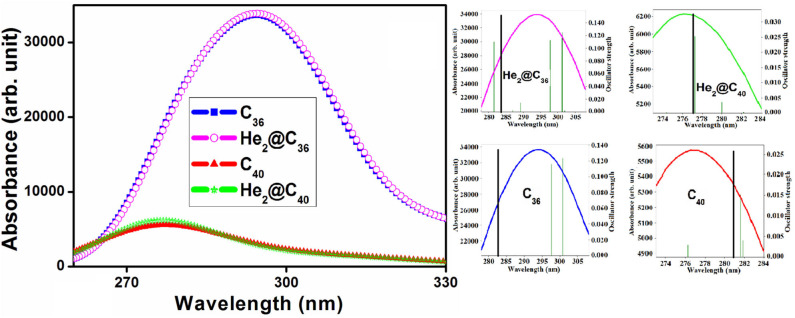
UV-visible absorption spectra of C_X_ and He_2_@C_X_ (*X* = 36, 40).

Table S1 provides the maximum absorption wavelength (λ_max_), corresponding transition energy (E_0_), highest oscillator strength (*f*_*max*_), and the major electronic transitions of the He_2_ encapsulated C_36_ and C_40_ fullerenes along with their empty counterparts. The associated absorption spectra are provided in Figure 5. Also, to get a clear view of the highest absorption peak and highest oscillator strength of both bare and He_2_-encapsulated fullerenes, a zoom plot is provided. We may see that both the He_2_-encaged fullerenes possess absorption maxima in the ultraviolet (UV) region of the spectra with their λ_max_ values of 283.652 and 277.090 nm for He_2_@C_36_ and He_2_@C_40_, respectively. One may observe that the highest oscillator strength of He_2_ confined C_36_ cage (0.1521) is much higher than that of C_40_ cage (0.0324). In addition, the corresponding λ_max_ of He_2_@C_36_ is red-shifted toward a higher wavelength compared to He_2_@C_40_. Again, from the absorption spectra, it should be pointed out that the maximum absorption of the empty fullerenes, i.e., C_36_ (282.572 nm) and C_40_ (280.669 nm), also occur in the UV region, having slight displacement of the peaks with respect to their He_2_-confined analogs. C_36_, He_2_@C_36_ has higher intensity of absorption compared to C_40_, He_2_@C_40_. This is because the former set (C_36_ and He_2_@C_36_) has recorded a much higher value of oscillator strength, which significantly increased its absorption maxima with respect to the later set (C_40_ and He_2_@C_40_). In addition, the increment in the oscillator strength of C_36_ and He_2_@C_36_ occurs may be due to higher value of transition dipole moment, which ultimately puts an impact on the intensity of the highest electronic transition (Wang et al., 2015).

The shift in the absorption maxima (either blue or red) depends on various factors; for example, the electronic structure (Yanagisawa et al., 2013; Giri et al., 2014), implementation of gas or solvent phase (Giri et al., 2014), orientation of embedded molecule inside any closed cage (Wang et al., 2018), substitution of donor or acceptors (Ma et al., 2019), and sometimes dependency on the method of calculation is also observed (Cárdenas-Jirón et al., 2017). Here, in this case, may be the electronic structure (symmetry, orientation of He_2_ inside the fullerenes) plays an important role in this shift. In addition, red shift of the absorption spectra is also associated with a smaller energy gap (HOMO-LUMO gap) (Giri et al., 2014). Thus, He_2_@C_36_ (3.028 eV) with comparatively lower value of energy gap than He_2_@C_40_ (3.980 eV), has its absorption maxima red shifted toward the greater wavelength region. These systems with their maximum absorption peak falling in the UV region are usable in designing UV light protection devices.

### Solar Cell Parameters

**Figure 6:**
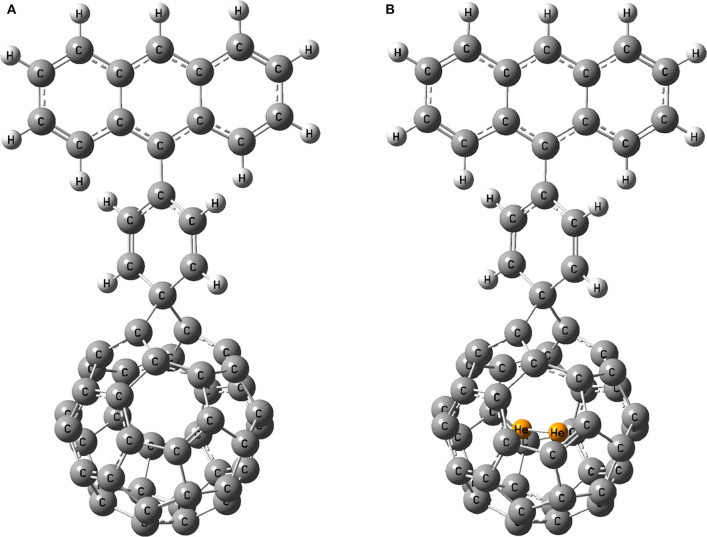
Optimized geometry of dyes **(A)** D1 and **(B)** D1@He_2_.

The most stable He_2_ encapsulated fullerene (He_2_@C_40_) among the two hosts is chosen as the acceptor for designing a solar cell device. To make a comparison, its free counterpart, pristine C_40_, is also taken as another acceptor. For the study, anthracene as donor and benzene as spacer are being used. For simplicity, we name the dye with acceptor as pristine C_40_ as D1 and that with He_2_@C_40_ as D1@He_2_. The optimized dyes are shown in Figure 6.

**Figure 7:**
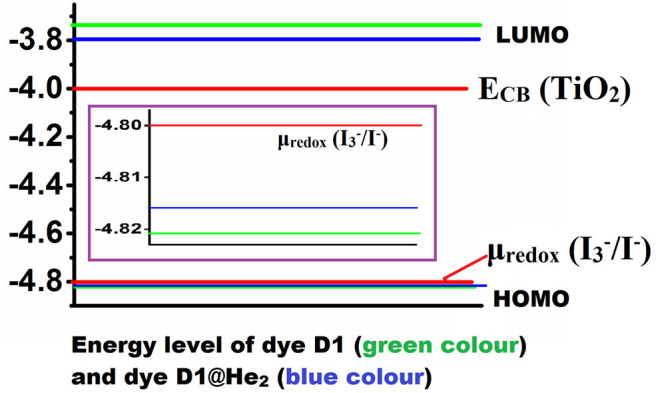
Energy level diagram of dyes D1 and D1@He_2_.

The energy levels of a dye molecule, which primarily indicates its HOMO and LUMO, play a very important role in its performance as a solar cell and the corresponding energy level diagram of the dyes is shown in Figure 7 (HOMO level of the dyes is zoomed). Eventually, the HOMO level of the dye must lie below the redox potential of $I_{3}^{-}/I^{-}$couple (μ_*redox*_ = −4.80 eV), while the LUMO level must be placed above the conduction band (CB) edge of TiO_2_ semiconductor (*E*_*CB*_ = −4.00 eV) (Qin et al., 2007). We found that the HOMO level of both the dyes lie just below the redox potential of $I_{3}^{-}/I^{-}$couple, with a magnitude of −4.821 eV (for D1) and −4.816 eV (for D1@He_2_), which confirms that charge regeneration of the dyes is possible. The LUMO value of dye, D1, is calculated to be −3.736 eV, while that of D1@He_2_ is −3.795 eV. This means the LUMO levels of both the dyes lie above the conduction band of TiO_2_, and thus electron injection from the excited dyes to the conduction band of the semiconductor (TiO_2_) will become easier. However, on comparing the HOMO and LUMO values of both the dyes, one may observe that the LUMO of D1 acquires some appreciable changes when He_2_ dimer is inserted in the acceptor (i.e., D1@He_2_ dye), but there is very less change in the HOMO energy. Therefore, it can be inferred that changing the acceptor can only affect the LUMO energy level of the dye. Moreover, dye D1 with comparatively higher LUMO value than D1@He_2_ will help to increase the open-circuit voltage, opening a path to improve the efficiency of the dye-sensitized solar cell. The energy gap of dyes D1 and D1@He_2_ are calculated to be 1.085 and 1.021 eV, respectively. Thus, dye D1@He_2_ with a relatively smaller energy gap and a comparatively higher light-harvesting efficiency will show a better result.

### Absorption Properties

**Figure 8:**
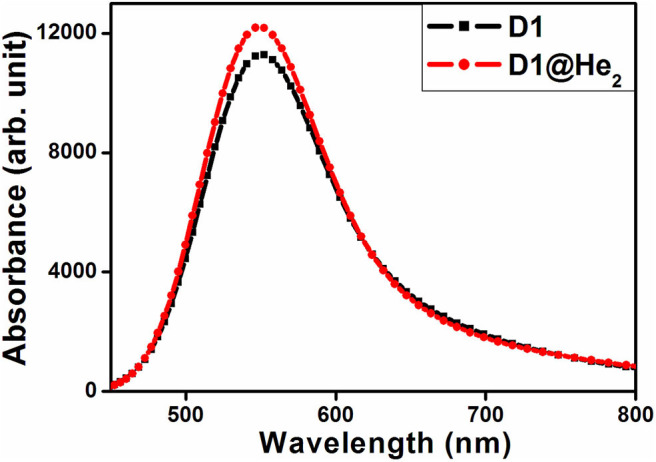
UV-visible absorption spectra of dyes D1 and D1@He_2_.

Here we have calculated the UV-visible absorption spectra of both the dyes (Figure 8) considering the lowest 40 transitions and results are provided in Table S2. One may observe that the maximum absorption wavelength corresponding to the highest oscillator strength (0.1456) of D1 dye falls at 547.126 nm with major transitions from HOMO → LUMO+3 (55%), HOMO → LUMO+5 (20%). For dye D1@He_2_, the highest oscillator strength increases to 0.1570 and there is a blue shift of the maximum absorption peak as compared to dye D1, located at 545.897 nm. There are two major transitions found for D1@He_2_ dye that take place from HOMO → LUMO+3 (53%) and HOMO → LUMO+5 (20%). Both the dyes show absorption in the visible region occurring at around 550 nm. From this study, we can infer that He_2_ dimer incorporation inside C_40_ increases the oscillator strength of dye D1. Thus, based on the study of absorption spectra, it is found that dye D1@He_2_ possesses a comparatively higher light-harvesting efficiency resulting a greater photocurrent response.

### Short-Circuit Current and Open-Circuit Voltage

The short circuit current (*J*_*SC*_) (Zhang et al., 2012) is given by the relation

(3) $$\begin{array}{r} J_{SC}=\overset{\;}{\underset{\lambda }{\int }}LHE\left(\lambda \right)\phi_{inject}\eta_{collect}d\lambda \end{array}$$

It greatly depends on the light-harvesting efficiency (*LHE*) and electron injection efficiency (ϕ_*inject*_). For the systems differing only in the choice of dyes, η_*collect*_ can be assumed constant.

(4) $$\begin{array}{r} LHE\text{ may be determined using }LHE=1-10^{-f_{\max}} \end{array}$$

On the other hand, ϕ_*inject*_ is related to the free energy of electron injection from the excited states of dye to the semiconductor surface (Δ*G*_*inject*_), which can be expressed as

(5) $$\begin{array}{r} \phi_{inject}\propto f\left({\Delta G}_{inject}\right) \end{array}$$

Δ*G*_*inject*_ can be estimated using the following equation (Katoh et al., 2004),

(6) $$\begin{array}{r} {\Delta G}_{inject}=E_{ox}^{dye*}-E_{CB}=E_{ox}^{dye}+E_{0}-E_{CB} \end{array}$$

where $E_{ox}^{dye*}$ represents the excited state oxidation potential of the dye, $E_{ox}^{dye}$ represents the ground state oxidation potential of the dye, *E*_0_ is the vertical transition energy corresponding to the maximum absorption wavelength of the dye, and *E*_*CB*_ refers to the conduction band edge of the semiconductor (TiO_2_).

The computed results of various quantities involved in the calculation of *J*_*SC*_ are provided in Table 3 for both the dyes. With the increase in the magnitude of $E_{ox}^{dye*}$, Δ*G*_*inject*_ increases, which ultimately increases the ϕ_*inject*_. Out of the two dyes, dye with the He_2_ dimer records the highest LHE value and thus will have a tendency to absorb more photons, which will lead to a higher magnitude of the short circuit current in comparison to dye D1. Also, according to a study (Islam et al., 2003), the electron injection efficiency of a dye is approximately equal to 1 when |Δ*G*_*inject*_| > 0.2. Here, we can see that both the dyes have a value of Δ*G*_*inject*_, which are much higher than 0.2. Thus, both of them possess sufficient driving force for electron injection to the semiconductor TiO_2_.

**Table 3:** Calculated solar cell parameters of dyes D1 and D1@He_2_.

**Dye**	**$E_{ox}^{dye}$ (eV)**	***E*_**0**_ (eV)**	$E_{ox}^{dye*}$ (eV)	**Δ*G*_***inject***_ (eV)**	**(*f_***max***_*)**	***LHE***	***eV*_***OC***_ (eV)**
D1	−4.821	2.266	−2.555	1.445	0.1456	0.2848	0.264
D1@He_2_	−4.816	2.271	−2.545	1.455	0.1570	0.3034	0.205

The open-circuit voltage (*V*_*OC*_) of a dye molecule can be evaluated approximately by finding the difference in energy between the LUMO energy level of the dye and the conduction band edge *E*_*CB*_ of the semiconductor substrate, which may be represented by the mathematical relation (Sang-aroon et al., 2012),

(7) $$\begin{array}{r} eV_{OC}=E_{LUMO}-E_{CB} \end{array}$$

The *eV*_*OC*_ values of the dyes are given in Table 3. The equation indicates that higher the magnitude of the LUMO level of the dye, the higher open-circuit voltage (*V*_*OC*_) it will generate. From Table 3 we can see that dye D1 records greater value of *eV*_*OC*_ than its He_2_ dimer encapsulated counterpart, which has a comparatively higher value of Δ*G*_*inject*_. This may be due to the fact that too high value of Δ*G*_*inject*_ leads to energy redundancy, making a fall in the value of *V*_*OC*_ (Li et al., 2017). Hence though the dye D1@He_2_ has a higher Δ*G*_*inject*_, but it possesses a smaller value of *V*_*OC*_ than dye D1.

### Non-linear Optical Properties

To explore the relationship between the efficiency of the dye molecules and their non-linear optical (NLO) properties, the isotropic polarizability of the dyes has been calculated. The response of any system when it is subjected to an external electric field is characterized by the study of NLO properties of that system (Deb et al., 2020). In case of dye D1, the isotropic polarizability is found to be 574.045 au, while for the dye D1@He_2_ the value increases to 577.945 au. This means that inclusion of a He_2_ dimer inside C_40_ acceptor improves the dye's polarizability. In addition, dyes having higher magnitudes of polarizability possibly will create strong interaction with its surroundings and will increase the local concentration of acceptor. Thus, the local concentration of the acceptor of the dye D1@He_2_, i.e., C_40_@He_2_ is increased on the semiconductor surface, which in turn will increase the possibility of this acceptor to perforate into the dye adsorption layer.

## Conclusion

Density functional theory has been implemented to assess the various properties of He_2_ dimer when encaged inside two fullerene cages, C_36_ and C_40_. Our study shows that when He_2_ is confined in the cages, its bond length considerably decreases. The compression in bond length is more when C_36_ holds the dimer as compared to that of its relatively larger counterpart, C_40_ fullerene. Non-covalent type of interaction exists between the He-He bond, whether in isolated or confined inside the cages, which is confirmed from the electron density analysis. From energy decomposition analysis it is observed that attractive interaction is found for He_2_ dimer, but after being encapsulated in the fullerenes, the interaction becomes repulsive. The presence of larger repulsive energy compared to the other attractive energy terms may be responsible for this change in the case of He_2_@C_36_ and He_2_@C_40_. Fullerene C_40_ bearing a larger cavity makes He_2_ incorporation energetically much easier than that of C_36_ as observed from the investigation of barrier crossing energy. Absorption spectra analysis of both the He_2_@C_X_ shows that they can be potentially used as UV light protectors since they possess absorption maxima in the UV region. Next, we designed a DSSC with free C_40_ and He_2_ confined C_40_ as acceptors. Charge regeneration and electron injection, which are the two most important qualities, are being fulfilled here by the dyes. In addition, both the dyes show an absorption peak in the visible region, which is another criterion for a DSSC. He_2_@C_40_, when used as acceptor, records the highest LHE value and thus will have a higher magnitude of *J*_*SC*_. NLO properties of the dyes are also calculated, and we found that the dye with He_2_-confined C_40_ acceptor has greater polarizability and thus will have higher possibility to perforate into the dye adsorption layer. This indicates that He_2_ incorporation inside fullerene really has a good effect on different properties.

## Data Availability Statement

The raw data supporting the conclusions of this article will be made available by the authors, without undue reservation.

## Author Contributions

DP has played the lead role in data collection, analysis, visualization, and writing original draft. HD has played a supporting role in data extraction and formal analysis. US has a lead role in supervision, funding and software, and supporting role in conceptualization. All authors contributed to the article and approved the submitted version.

## Conflict of Interest

The authors declare that the research was conducted in the absence of any commercial or financial relationships that could be construed as a potential conflict of interest.
