# Supplementary material for: Confinement Effects of a Noble Gas Dimer Inside a Fullerene Cage: Can It Be Used as an Acceptor in a DSSC?
Source: Front Chem. 2020 Aug 6;8:621. doi: 10.3389/fchem.2020.00621 (PMC7424018; doi:10.3389/fchem.2020.00621)
Supplement: Supplementary file 1 [file Data_Sheet_1.docx]

**Supplementary Material**

**
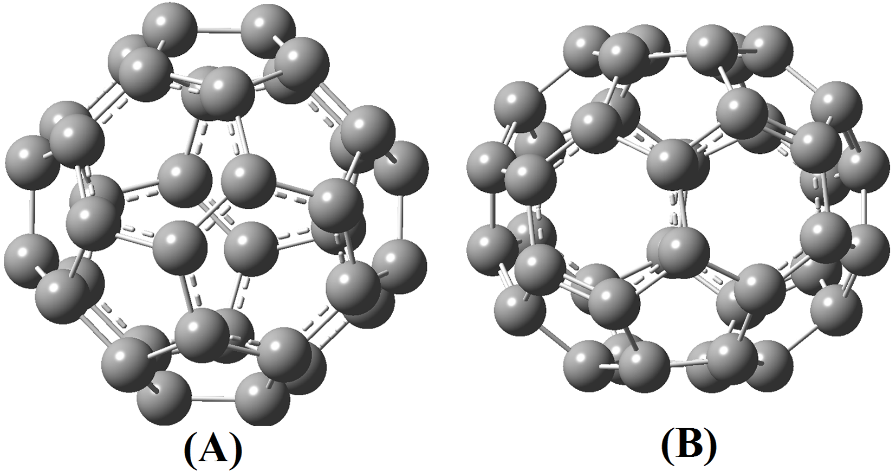
**

**Figure S1 |** Optimized geometry of **(A)** C_36_ and **(B)** C_40_.

**
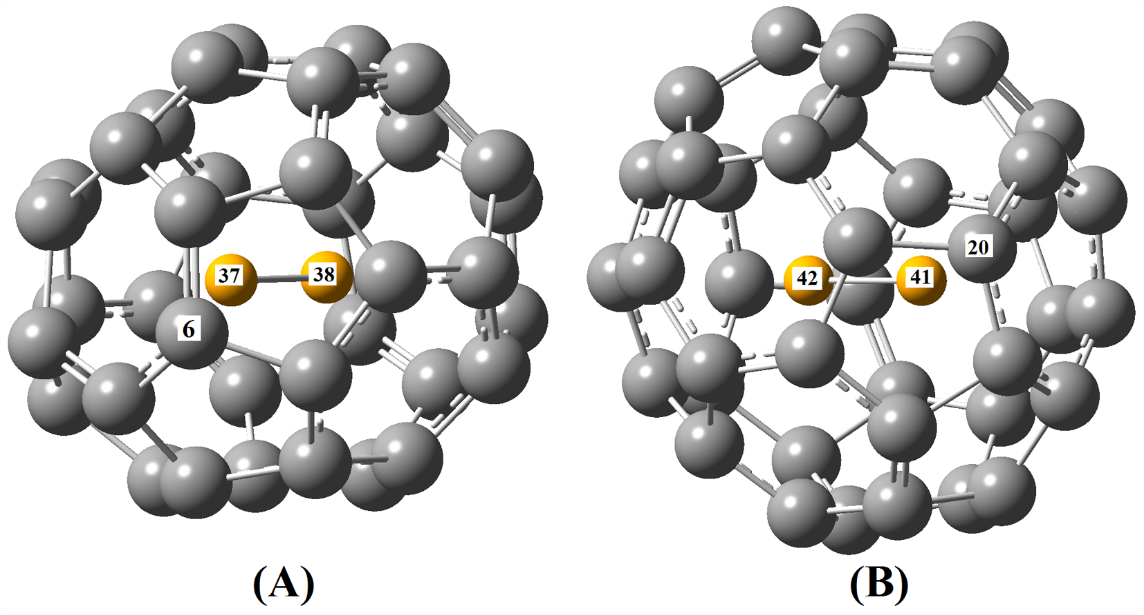
**

**Figure S2 |** Labelled figure of **(A)** He_2_@C_36_ and **(B)** He_2_@C_40_.

**Table S1 |** Absorption wavelength (λ_max_), transition energy (E_0_), highest oscillator strength (*f_max_*) and the major electronic transitions of C_X_ and He_2_@C_X_ (X = 36, 40).

| **System** | **λ_max_ (nm)** | **E_0_ (eV)** | **(*f_max_*)** | **Major transitions** |
| --- | --- | --- | --- | --- |
| **C_36_** | 282.572 | 4.388 | 0.1337 | HOMO→LUMO+8 (71%)  HOMO-7→LUMO+2 (7%) |
| **C_36_@He_2_** | 283.652 | 4.371 | 0.1521 | HOMO→LUMO+8 (63%)  HOMO-7→LUMO+2 (12%) |
| **C_40_** | 280.869 | 4.414 | 0.0259 | HOMO-10→LUMO (36%)  HOMO→LUMO+5 (20%) |
| **C_40_@He_2_** | 277.090 | 4.475 | 0.0324 | HOMO-10→LUMO (36%)  HOMO→LUMO+6 (21%) |

**Table S2 |** Transition wavelength (λ_max_) and major electronic transitions of D1 and D1@He_2_.

| **System** | **λ_max_ (nm)** | **Major transitions** |
| --- | --- | --- |
| **D1** | 547.126 | HOMO → LUMO+3 (55%)  HOMO → LUMO+5 (20%) |
| **D1@He_2_** | 545.897 | HOMO → LUMO+3 (53%)  HOMO → LUMO+5 (20%) |
